# Mex3a interacts with LAMA2 to promote lung adenocarcinoma metastasis via PI3K/AKT pathway

**DOI:** 10.1038/s41419-020-02858-3

**Published:** 2020-08-13

**Authors:** Jinghui Liang, Haixia Li, Jingyi Han, Jin Jiang, Jiang Wang, Yongmeng Li, Zitong Feng, Renchang Zhao, Zhenguo Sun, Bin Lv, Hui Tian

**Affiliations:** 1 Department of Thoracic Surgery, Qilu Hospital, Cheeloo College of Medicine, Shandong University, 250012 Jinan, Shandong China; 2 School of Basic Medical Sciences of Shandong University, 250012 Jinan, China; 3 Weifang People’s Hospital, 261000 Weifang, China; 4 Department of General Surgery, Cheeloo College of Medicine, Shandong University, 250012 Jinan, Shandong China; 5 School of Medicine, Shandong University, 250012 Jinan, China

**Keywords:** Cancer genomics, Non-small-cell lung cancer

## Abstract

Lung adenocarcinoma (LUAD) is the main subtype of lung cancer. In this study, we found that RBP Mex3a was significantly upregulated in LUAD tissues and elevated Mex3a expression was associated with poor LUAD prognosis and metastasis. Furthermore, we demonstrated that Mex3a knockdown significantly inhibited LUAD cell migration and invasion in vitro and metastasis in nude mice. Transcriptome sequencing indicated that Mex3a affected gene expression linked to ECM-receptor interactions, including laminin subunit alpha 2(LAMA2). RNA immunoprecipitation (RIP) assay revealed Mex3a directly bound to LAMA2 mRNA and Mex3a increased the instability of LAMA2 mRNA in LUAD cells. Furthermore, we discovered that LAMA2 was surprisingly downregulated in LUAD and inhibited LUAD metastasis. LAMA2 knockdown partially reverse the decrease of cell migration and invasion caused by Mex3a knockdown. In addition, we found that both Mex3a and LAMA2 could influence PI3K-AKT pathway, which are downstream effectors of the ECM-receptor pathway. Moreover, the reduced activation of PI3K-AKT pathway in caused by Mex3a depletion was rescued by LAMA2 knockdown. In conclusion, we demonstrated that Mex3a downregulates LAMA2 expression to exert a prometastatic role in LUAD. Our study revealed the prognostic and prometastatic effects of Mex3a in LUAD, suggesting that Mex3a can serve as a prognostic biomarker and a target for metastatic therapy.

## Introduction

Lung adenocarcinoma (LUAD) is the main malignant tumor of the lung and is one of the most common causes of cancer-related death worldwide. Despite tremendous efforts to improve early detection and develop new treatment strategies, patients with LUAD still face a high incidence of postoperative recurrence and unsatisfactory survival rates. Therefore, it is important to elucidate the potential oncogenic molecular mechanisms to develop new therapies targeting LUAD.

As RNA-binding protein, Mex3a gene is one of four human homologous Mex3 genes. Four Mex3 homologous genes have been discovered, namely: Mex3A, Mex3B, Mex3C, and Mex3D. The four Mex3 proteins in humans have similar specific structures that bind RNA and are involved in the regulation of RNA metabolism^1^. Studies have shown that Mex3A is part of newly discovered processing bodies^1^. Processing bodies is an important site in the post-transcriptional regulation of mRNA and plays a crucial role in the regulation of gene expression. Studies in recent years have shown that Mex3a may be closely linked to cancer. Pereira et al. first predicted that Mex3a inhibited the expression of CDX2, indicating that the gene may have carcinogenic effects^2^. In mice, Mex3a labeled Lgr5+intestinal stem cell population^3^. Recent studies have reported that Mex3a is overexpressed in cancers like bladder urothelial carcinoma and Wilm’s tumor meanwhile Mex3a gene is closely related to the proliferation, apoptosis, and metastasis of gastric cancer cells^4–6^. But little is known about the expression and function of Mex3a in lung adenocarcinoma.

As large extracellular glycoproteins, laminins (LMs) involved in several biological processes including cellular interactions, self-polymerization and binding with other extracellular matrix (ECM) proteins are that are important components of basement membranes^7–9^. LAMA2 gene encodes an alpha 2 chain, which constitutes one of the subunits of laminin 2. Loss of LAMA2 can lead into muscular dystrophy^10–12^. And decreased expression of LAMA2 caused by promoter hypermethylation has been confirmed in various cancers, including invasive PiNETs, colon, and bladder cancers, which indicate LAMA2 is a suppressor gene^13,14^. LAMA2 can also modulate PTEN to affect PI3K/AKT pathway^13^. However, the role of LAMA2 in lung adenocarcinoma remains unknown.

In this study, we first identified the RBP Mex3a as a metastasis promoter and LAMA2 as a metastasis inhibitor in LUAD. More interestingly, we found that knockdown LAMA2 could reverse Mex3a-knockdown-induced metastasis in LUAD. Therefore, our findings characterized a novel post-transcriptional mechanism through Mex3a-mediated LAMA2 transcript instability and could provide theoretical rationality for the potential clinical application value of such therapeutic targets for precise treatment of LUAD individuals.

## Results

### Mex3a expression was frequently upregulated in LUAD tissues and predicted overall survival time in LUAD patients

**Fig. 1:**
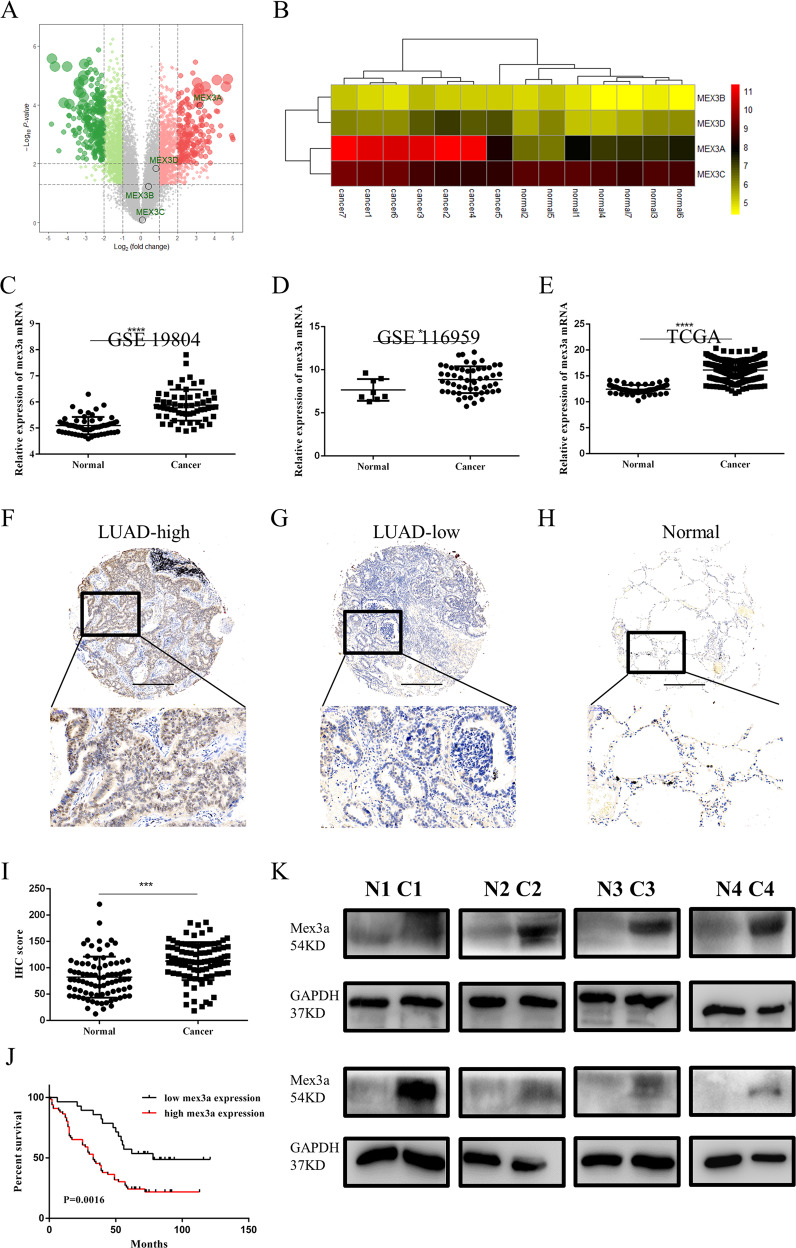
Expression and prognostic significance of mex3a in LUAD. **a** Volcano of altered genes in the mex3a family in seven pairs of LUAD tissues and tumor-adjacent tissues from transcriptome Microarray sequencing. **b** Heatmap of altered genes in the mex3a family in LUAD. High and low expression levels are indicated in red and yellow. **c**–**e** mex3a mRNA expression analysis in LUAD and nontumor tissues in GEO (GSE 19804 (**c**) and GSE 116959 (**d**)) and TCGA(E)datasets. **f**–**h** Representative IHC images of mex3a expression of high expression (**f**) and low expression (**g**) of LUAD and para-tumor (**h**). **i** IHC score for mex3a in the tissue microarray. **j** Kaplan–Meier analysis of LUAD cohorts based on predictive survival analysis. **k** mex3a protein expression was detected by western blot in eight paired LUAD tissue samples. *P < 0.05, **P < 0.01, ***P < 0.001, and ****P < 0.0001.

First, we used seven pairs frozen LUAD tissues and adjacent normal tissues to screen potential biomarkers by transcriptome microarray (GSE 140797). Based on the microarray results, we found the Mex3a was significantly overexpressed among Mex3 family in LUAD tissues than in adjacent normal tissues (Fig. 1a, b). To investigate the potential role of Mex3a in human LUAD pathogenesis, we firstly carried out an analysis of GEO LUAD databases (GSE 19804 and GSE 116959) and TCGA LUAD database. Similar to our microarray results, we found that the mRNA expression of Mex3a was upregulated in LUAD tissues compared to adjacent normal tissues (Fig. 1c–e). Subsequently, we wondered whether Mex3a was elevated at the protein level in LUAD tissues. We carried out IHC staining to analyze the protein expression of Mex3a in 94-paired LUAD patients (Fig. 1f–h). IHC score indicated that Mex3a expression was significantly increased in LUAD tissues in contrast to that in adjacent normal tissues (Fig. 1i). Similarly, Mex3a protein expression was higher in eight LUAD samples than in adjacent nontumor tissues (Fig. 1k).

We then analyzed the relation between the clinicopathological parameters and Mex3a expression in LUAD patients. The results showed that 26 (27.7%) and 68 (72.3%) patients had low and high Mex3a expression, respectively (Table 1), and high Mex3a expression was significantly related to lymph node status (*P* = 0.038, Table 1), TNM stage (*P* = 0.007, Table 1), and pathology stage (*P* = 0.028, Table 1), but not to age, gender, or metastasis stage (Table 1). Furthermore, Kaplan–Meier survival analysis indicated that patients with higher Mex3a expression appeared to have a shorter OS (Fig. 1j, *P* < 0.05) compared with those patients who expressed lower levels of Mex3a. Similarly, poor OS (*n* = 673) and PFS (*n* = 443) from K–M Plotter were observed in the highly expressed Mex3a in LUAD patients (Supplementary Fig. 1A, B). Taken together, our data imply the oncogenic role of Mex3a in LUAD.

**Table 1:** Correlation between Mex3a expression and clinicopathological features in LUAD.

		Mex3a expression		
Factors	Sample	Low expression	High expression	*P* value^a^
Age^b^				0.554
<60	43 (45.7)	11	32	
≥60	51 (54.3)	17	34	
Gender				0.436
Male	53 (56.4)	18	35	
Female	41 (43.6)	10	31	
pT status				0.805
T1	20 (21.3)	7	13	
T2	50 (53.2)	16	34	
T3	18 (19.1)	4	14	
T4	6 (6.4)	14	5	
pN status				**0.038**
N0	42 (44.7)	17	25	
N1	17 (18.1)	6	11	
N2	35 (37.2)	5	30	
pM status				1
M0	93 (98.9)	28	65	
M1	1 (1.1)	0	1	
TNM stage				**0.007**
I	30 (31.9)	15	15	
II	20 (21.3)	6	14	
III + IV	44 (46.8)	7	37	
Pathology stage				**0.028**
I	11 (11.7)	7	4	
II	52 (55.3)	15	37	
III	31 (33)	6	25	
Event				**0.018**
Alive	29 (30.9)	14	15	
Dead	65 (69.1)	14	51	
Mex3a expression				
Low expression	26 (27.7)			
High expression	68 (72.3)			

^a^The Pearson Chi-squared test or Fisher’s exact test was used for statistical analysis.

^b^The median age at diagnosis is 60 years in LUAD patients. Samples are divided into two groups based on the median age.

The statistical significance bold values *p* is less than 0.05.

### Mex3a promoted LUAD cell proliferation, migration, and invasion in vitro

**Fig. 2:**
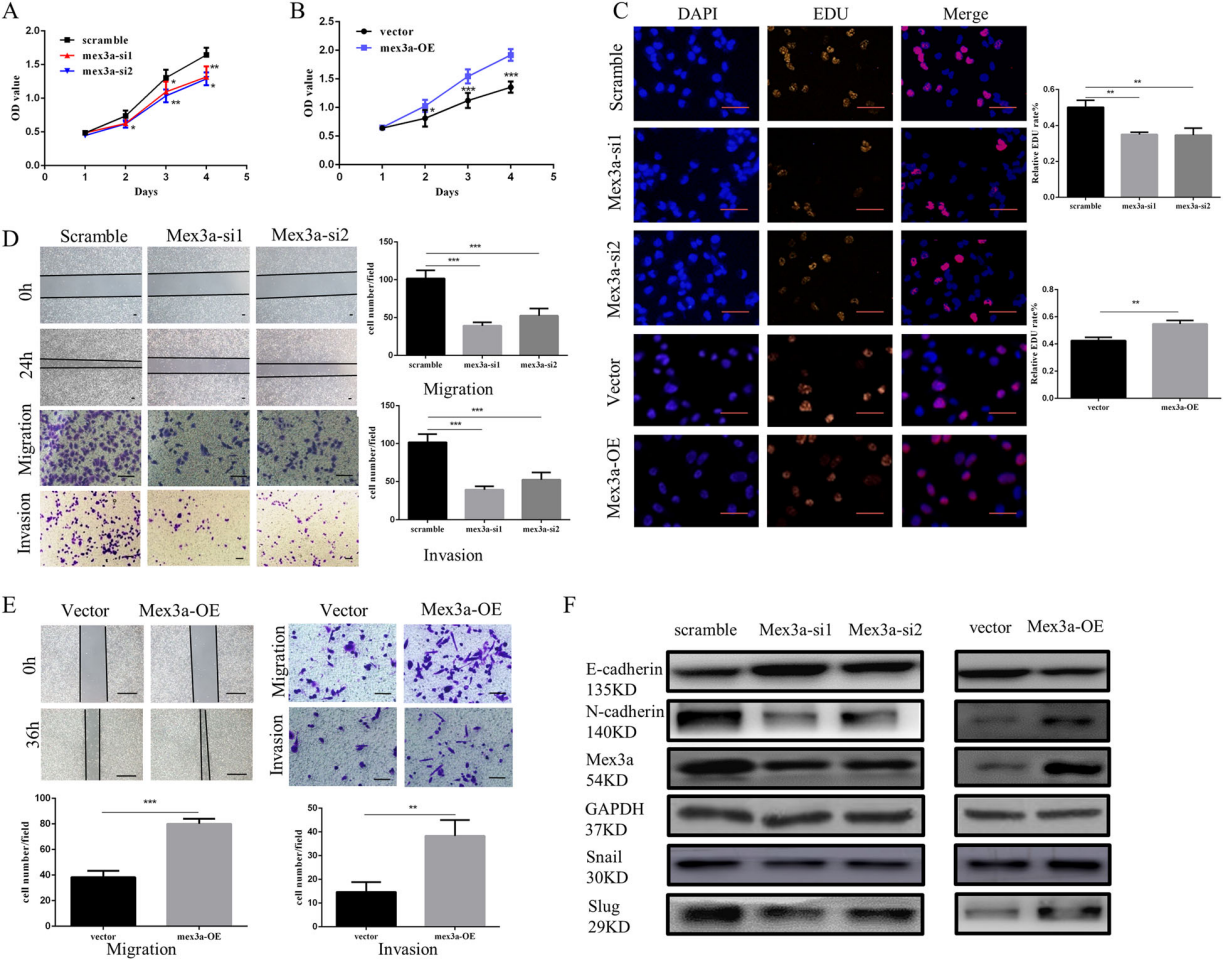
Mex3a promotes LUAD cell proliferation, migration, and invasion in vitro. **a**–**c** CCK8 and EDU assays were performed to identify proliferation after mex3a knockdown in H1299 cell (**a**, **c**) and mex3a overexpression in PC9 cell (**b**, **c**) Data are presented as the mean ± S.D. *n* = 3. **d**, **e** Wound healing, migration, and invasion assays were performed to identify metastasis ability after mex3a knockdown in H1299 cell (**d**) and mex3a overexpression in PC9 cell. Data are presented as the mean ± S.D. *n* = 3 (**e**). **f** Changes in the expression of the EMT biomarkers E-cadherin, N-cadherin, Snail, and Slug after mex3a knockdown and overexpression were detected by western blot. *P < 0.05, **P < 0.01, ***P < 0.001, and ****P < 0.0001.

We found H1299 and A549 cell had higher expression of Mex3a, and PC9 cell had lower expression of Mex3a among seven cell lines (Supplementary Fig. 1C, D). Then we choose H1299 cell and PC9 cell to explore the effect of Mex3a on LUAD cell. We established Mex3a-knockdown H1299 cell with two sequences of siRNA and Mex3a overexpression PC9 cell (Supplementary Fig. 1E, F). Mex3a knockdown altered the proliferation rate and the ability of migration and invasion of H1299 cell (Fig. 2a, c, d). Similarly, Mex3a overexpression promoted the proliferation rate and the ability of migration and invasion of PC9 cell (Fig. 2b, c, e). Besides, levels of EMT-related gene N-cadherin, Snail, and Slug were downregulated and E-cadherin was upregulated in Mex3a-knockdown H1299 cell. The opposite results were showed in Mex3a overexpression PC9 cell (Fig. 2g).

### Mex3a promoted LUAD cell proliferation, migration, and invasion in vivo

**Fig. 3:**
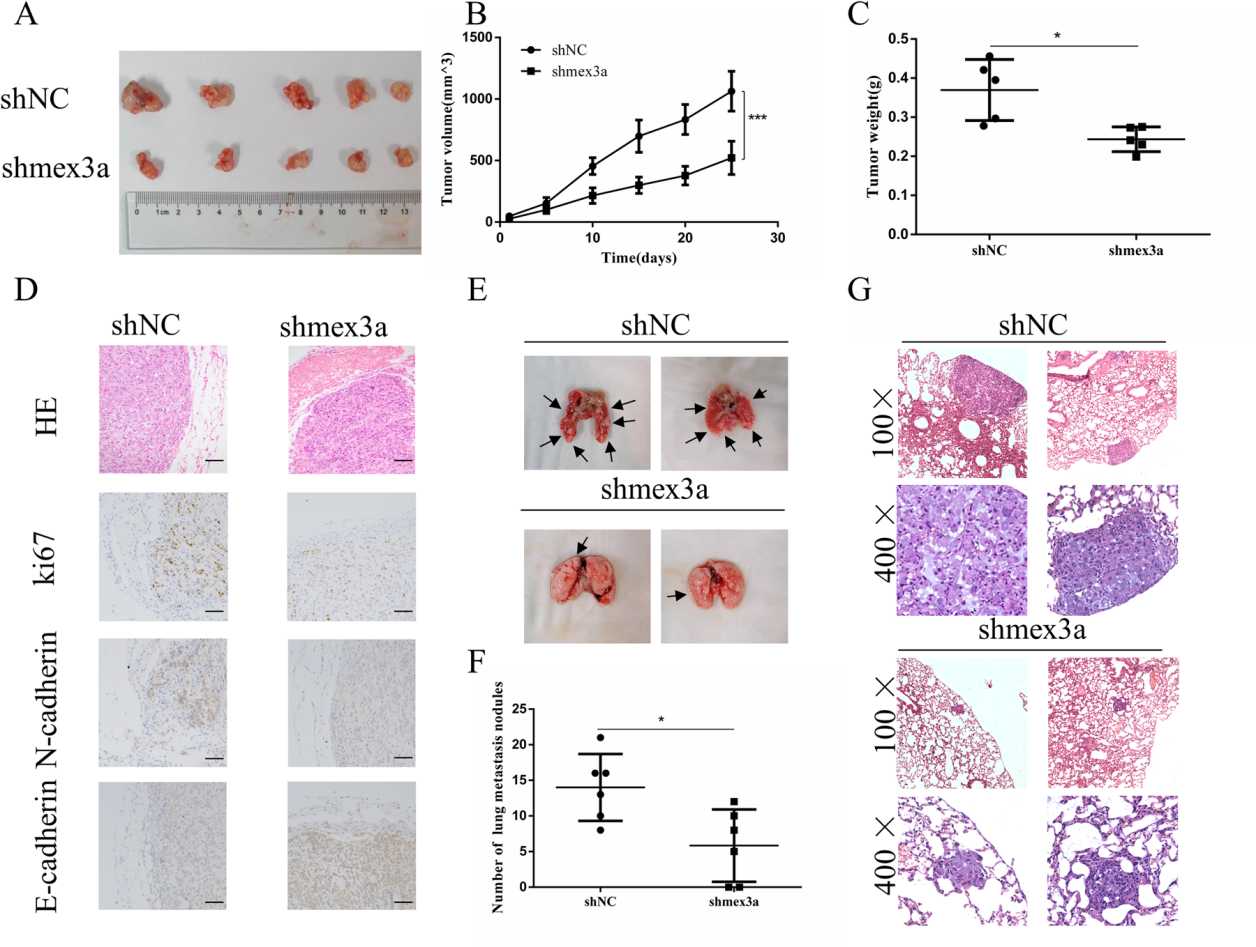
Mex3a promotes LUAD cell proliferation and metastasis in vivo. **a** Representative images of tumor from different treatment groups 5 weeks after tumor injections. **b**, **c** The volumes (**b**) and weights (**c**) were lower for xenograft tumors with mex3a knockdown than for xenograft tumors with scrambled shRNA. **d** Representative images of IHC staining of Ki67, E-cadherin, and N-cadherin in xenograft tumors. **e** Representative images of lung metastasis. **f** Bar chart of lung metastasis nodules in shNC and shmex3a groups. **g** Representative images of HE staining of lung metastasis. *P < 0.05, **P < 0.01, ***P < 0.001, and ****P < 0.0001.

Furthermore, we performed in vivo experiments to evaluate the oncogenic effect of mex3a. We first established A549 and H1299 shMex3a cell line (Supplementary Fig. 1G, H). An engrafted tumor mouse model was established by implanted the H1299 cells subcutaneously in nude mice (Fig. 3a). the mice that received Mex3a-knockdown H1299 cell resulted in a significant inhibition of tumor growth and tumor weight (Fig. 3b, c). Therefore, IHC staining proved that the expression of Ki67 and N-cadherin in the shMex3a group was significantly lower than that in the control group, while the expression of E-cadherin in the shMex3a group was significantly higher than that in the control group (Fig. 3d). We performed tail vein injection with Mex3a-knockdown H1299 cells to establish tumor metastasis mouse models (Fig. 3e). Mex3a knockdown decreased the number of metastasis lesions (Fig. 3f). Tumor metastasis was confirmed by HE staining (Fig. 3g). Therefore, both in vitro and in vivo data supported the metastatic effect of Mex3a in LUAD.

### Mex3a changed the expression of genes associated with ECM-receptor interaction and PI3K-AKT pathway

**Fig. 4:**
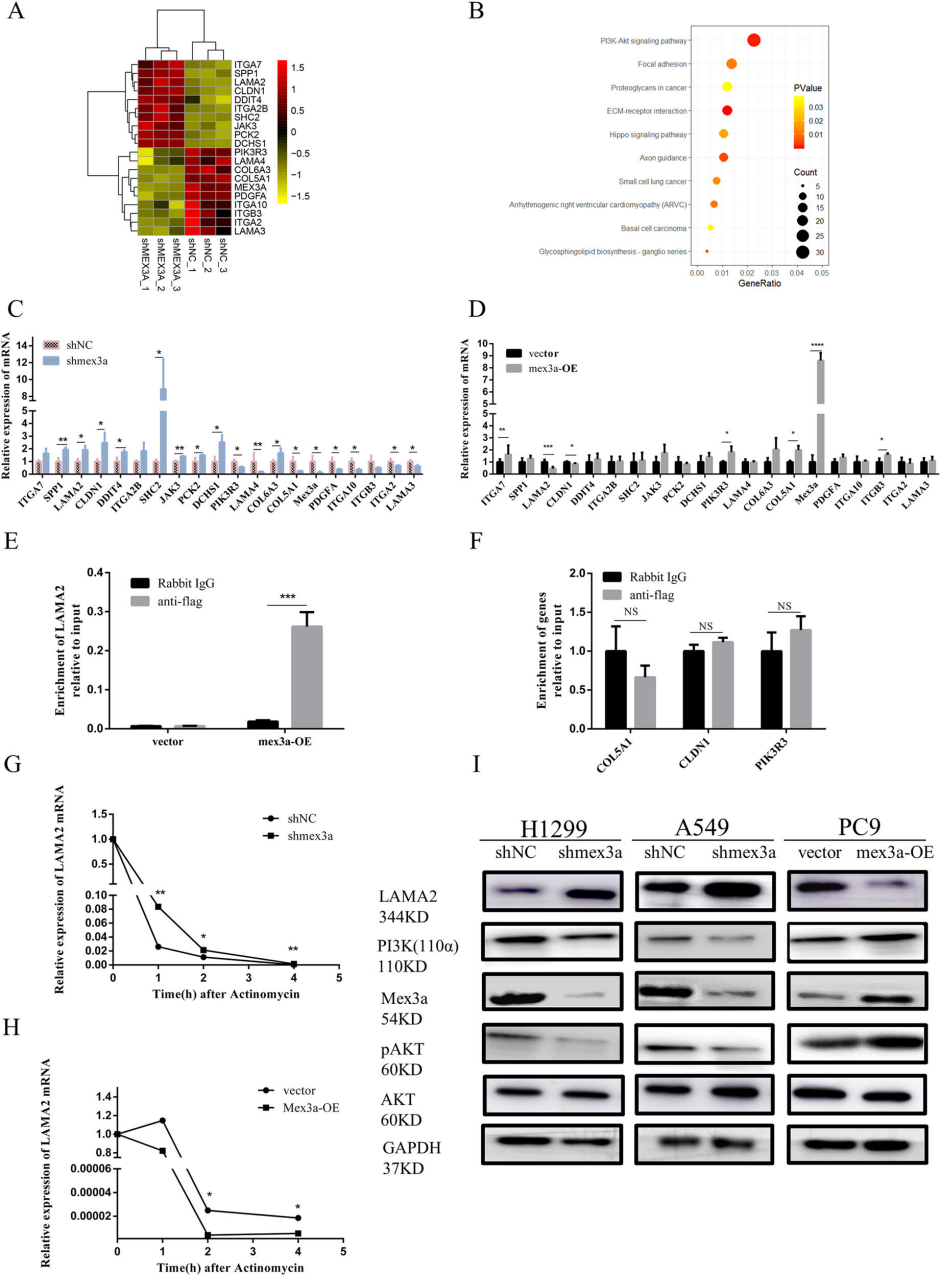
Mex3a change the expression of genes associated with ECM-receptor interaction and PI3K-AKT pathway. **a** Heatmap of distinctly dysregulated mRNAs in stable H1299 cell lines (scrambled shRNA vs shmex3a) identified from transcriptome sequencing by hierarchical clustering. High and low expression levels are indicated in red and yellow, respectively. **b** Signaling pathways of altered genes after mex3a knockdown in H1299 cells detected by microarray analysis. Statistical significance is indicated by different colors. The size of the circle represents the number of genes. **c** The mRNA levels of 20 candidate genes in H1299 cells with mex3a knockdown were detected by qRT-PCR. **d** The mRNA levels of 20 candidate genes in PC9 cells with mex3a overexpression were detected by qRT-PCR. Data are presented as the mean ± S.D. *n* = 3. **e**, **f** RIP experiment showed that anti-flag antibody could not precipitate COL5A1, CLDN1, and PIK3R3 but LAMA2 in Mex3a overexpression of PC9 cells. Data are presented as the mean ± S.D. *n* = 3. **g**, **h** Knockdown and overexpression of mex3a in H1299 and PC9 cell were treated with actinomycin D (5 μg/mL). The cells were harvested at the indicated times after actinomycin D was added. I. LAMA2 and PI3K-AKT pathway expression was regulated by mex3a. *P < 0.05, **P < 0.01, ***P < 0.001, and ****P < 0.0001.

To gain insights into molecular mechanism of Mex3a-induced metastasis of LUAD, transcriptome sequencing was performed by using Mex3a-silenced H1299 cells and control cells. Pathway analysis showed that Mex3a knockdown had important effects on genes that are mainly related to ECM-receptor interactions and PI3K-AKT pathway (Fig. 4b). The ECM-receptor interaction and PI3K-AKT pathway were composed of many well-known genes that play a key role in cell movement and cancer metastasis, which is consistent with the metastasis-promoting effect of Mex3a^15–17^. Then we selected several significantly altered, ECM-receptor interactions and PI3K-AKT pathway involved genes (fold-change > 1.5) (Fig. 4a).

### LAMA2 was a downstream target of Mex3a in LUAD

**Fig. 5:**
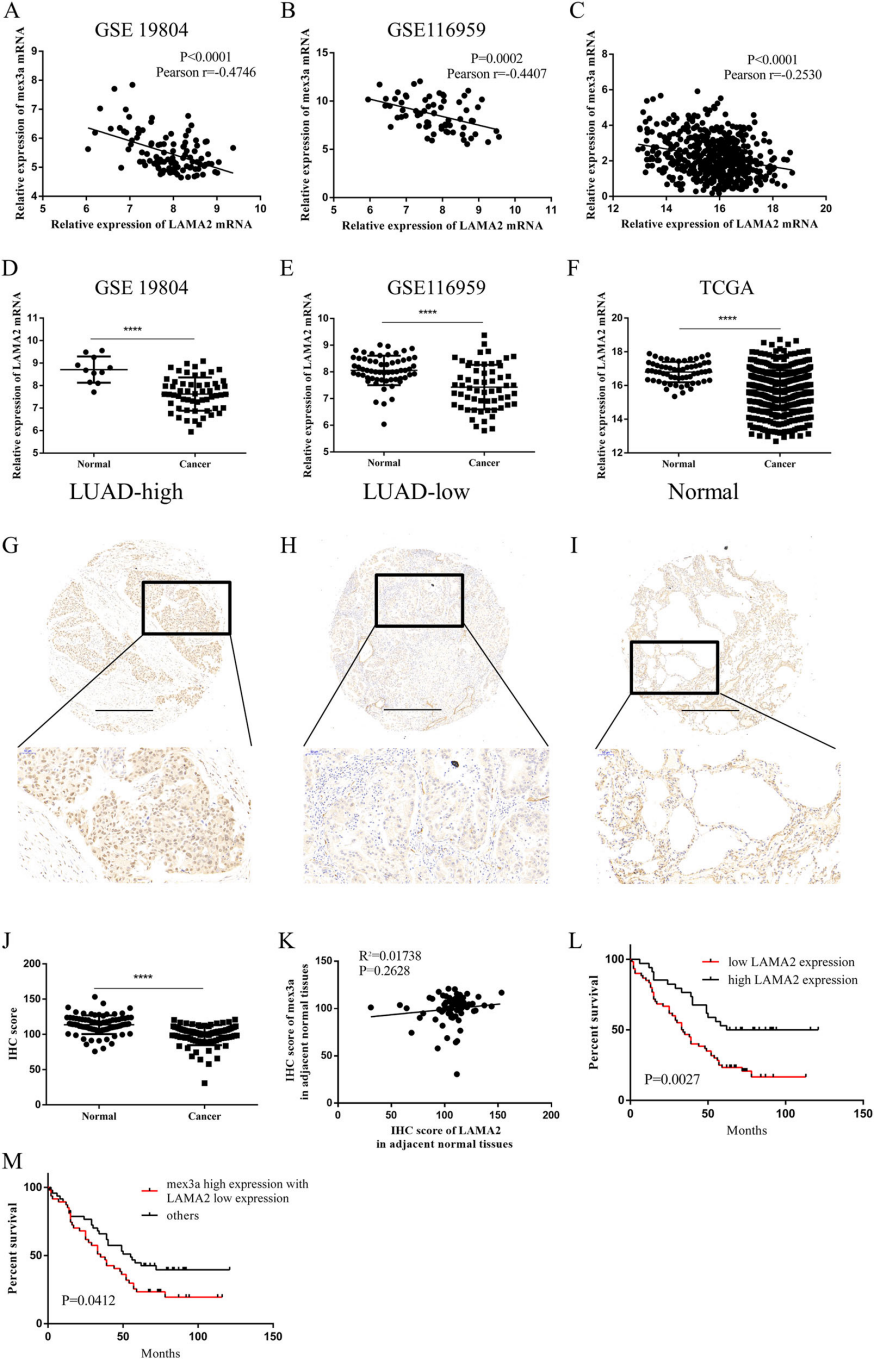
LAMA2 is a downstream target of mex3a in LUAD. **a**–**c** The association between relative mex3a and LAMA2 mRNA levels in GEO (GSE 19804 (**a**) and GSE 116959 (**b**)) and TCGA (**c**) datasets was analyzed according to the Pearson-correlation factor. **d**, **f** LAMA2 mRNA expression analysis in LUAD and nontumor tissues in GEO (GSE 19804 (**d**) and GSE 116959 (**e**)) and TCGA (**f**) datasets. **g**–**i** Representative IHC images of LAMA2 expression of high expression (**g**) and low expression (**h**) of LUAD and para-tumor (**i**). **j** IHC score for LAMA2 in the tissue microarray. **k** Correlation between Mex3a and LAMA2 in adjacent normal tissues. **l** Overall survival curves for patients with LUAD were stratified according to LAMA2 expression. **m** Overall survival curves for patients with LUAD were stratified according to patients with high expression of mex3a and low expression of LAMA2, and other patients. *P < 0.05, **P < 0.01, ***P < 0.001, and ****P < 0.0001.

We performed qRT-PCR analysis to confirmed consistency of 20 genes in Mex3a overexpression and Mex3a-knockdown cell. The results suggested LAMA2, CLDN1, PIK3R3, and COL5A1 as possible targets of Mex3a (Fig. 4c, d). Then we used RIP assays to find out whether Mex3a interacted with those genes. We chose Mex3a overexpressing PC9 cell, which have flag label inserted in plasmid. Interestingly, the LAMA2 mRNA was significantly enriched in anti-flag sample compared with rabbit IgG sample in PC9 cell while CLDN1, PIK3R3, and COL5A1 failed (Fig. 4e, f). We next examined the effects of Mex3a on LAMA2 mRNA stability by actinomycin D experiment. LAMA2 mRNA decayed more rapidly in the presence of Mex3a and more slowly in the absence of Mex3a (Fig. 4g, h). Western blotting further confirmed that the expression of LAMA2 and key molecule in PI3K-AKT pathway was regulated by Mex3a (Fig. 4i). Then we evaluated the correlation between Mex3a and LAMA2 mRNA in GEO (GSE 19804 and GSE 116959) and TCGA datasets (Fig. 5a–c). Strikingly, all the result suggested LAMA2 was negative to Mex3a in LUAD. Therefore, further experiments focused on whether Mex3a function was the result of regulation of LAMA2 expression.

Because the function of LAMA2 was unclear in LUAD, so we determined first to investigate LAMA2 expression in LUAD and adjacent normal tissues using transcriptome data downloaded from GEO (GSE 19804 and GSE 116959) and TCGA datasets and discovered that LAMA2 exhibited lower expression in LUAD tissues than in adjacent normal tissues (Fig. 5d–f). Then we carried out IHC staining to analyze expression of LAMA2 in same 94 LUAD patients (Fig. 5g–i). We found LUAD tissue expressed lower LAMA2 expression than para-tumor tissue (Fig. 5j). The correlation analysis suggested that LAMA2 expression was significantly negative related with Mex3a in LUAD (Table 2). We failed to discover the relationship between Mex3a and LAMA2 in adjacent normal tissues (Fig. 5k). What’s more, we evaluated the prognostic values of LAMA2 in LUAD with the Kaplan–Meier analysis. Low LAMA2 expression was also positively correlated with low OS in LUAD (Fig. 5l). Similarly, poor OS and PFS from K–M Plotter were observed in the lowly expressed LAMA2 in LUAD patients (Supplementary Fig. 1I, J). We combined the Mex3a and LAMA2 expression and evaluated the prognostic value of the patients with high expression of Mex3a and low expression of LAMA2. Patients with high expression of Mex3a and low expression of LAMA2 tended to have worse prognosis than other patients (Fig. 5m), suggesting that co-expression of Mex3a and LAMA2 may be a more sensitive factor of LUAD, and low LAMA2 expression was associated with and advanced TNM stage (Table 3).

**Table 2:** Correlation analysis between mex3a and LAMA2 in LUAD tissue in microarray.

Correlation analysis	Mex3a low expression	Mex3a high expression	Total	Pearson
LAMA2 low expression	10	50	60	
LAMA2 high expression	16	18	34	*R* = −0.3265
Total	26	68	94	*P* = 0.0013

**Table 3:** Correlation between LAMA2 expression and clinicopathological features in LUAD.

		LAMA2 expression		
Factors	Sample	Low expression	High expression	*P* value^a^
Age^b^				0.6669
<60	43 (47.3)	26	17	
≥60	51 (52.7)	34	17	
Gender				1
Male	53 (56.4)	34	19	
Female	41 (43.6)	26	15	
pT status				0.3942
T1	20 (21.3)	12	8	
T2	50 (53.2)	34	16	
T3	18 (19.1)	9	9	
T4	6 (6.4)	5	1	
pN status				0.1123
N0	42 (44.7)	24	18	
N1	17 (18.1)	9	8	
N2	35 (37.2)	27	8	
pM status				1
M0	93 (98.9)	59	34	
M1	1 (1.1)	1	0	
TNM stage				**0.0459**
I	30 (31.9)	17	13	
II	21 (22.3)	‘10	11	
III + IV	43 (45.8)	33	10	
Pathology stage				0.7900
I	11 (11.7)	6	5	
II	52 (55.3)	34	18	
III	31 (33)	20	11	
Event				**0.0047**
Alive	29 (30.9)	12	17	
Dead	65 (69.1)	48	17	
LAMA2 expression				
Low expression	60 (63.8)			
High expression	34 (36.2)			

^a^The Pearson Chi-squared test or Fisher’s exact test was used for statistical analysis.

^b^The median age at diagnosis is 60 years in LUAD patients. Samples are divided into two groups based on the median age.

The statistical significance bold values *p* is less than 0.05.

### Mex3a activated the PI3K/AKT signaling pathways through LAMA2

**Fig. 6:**
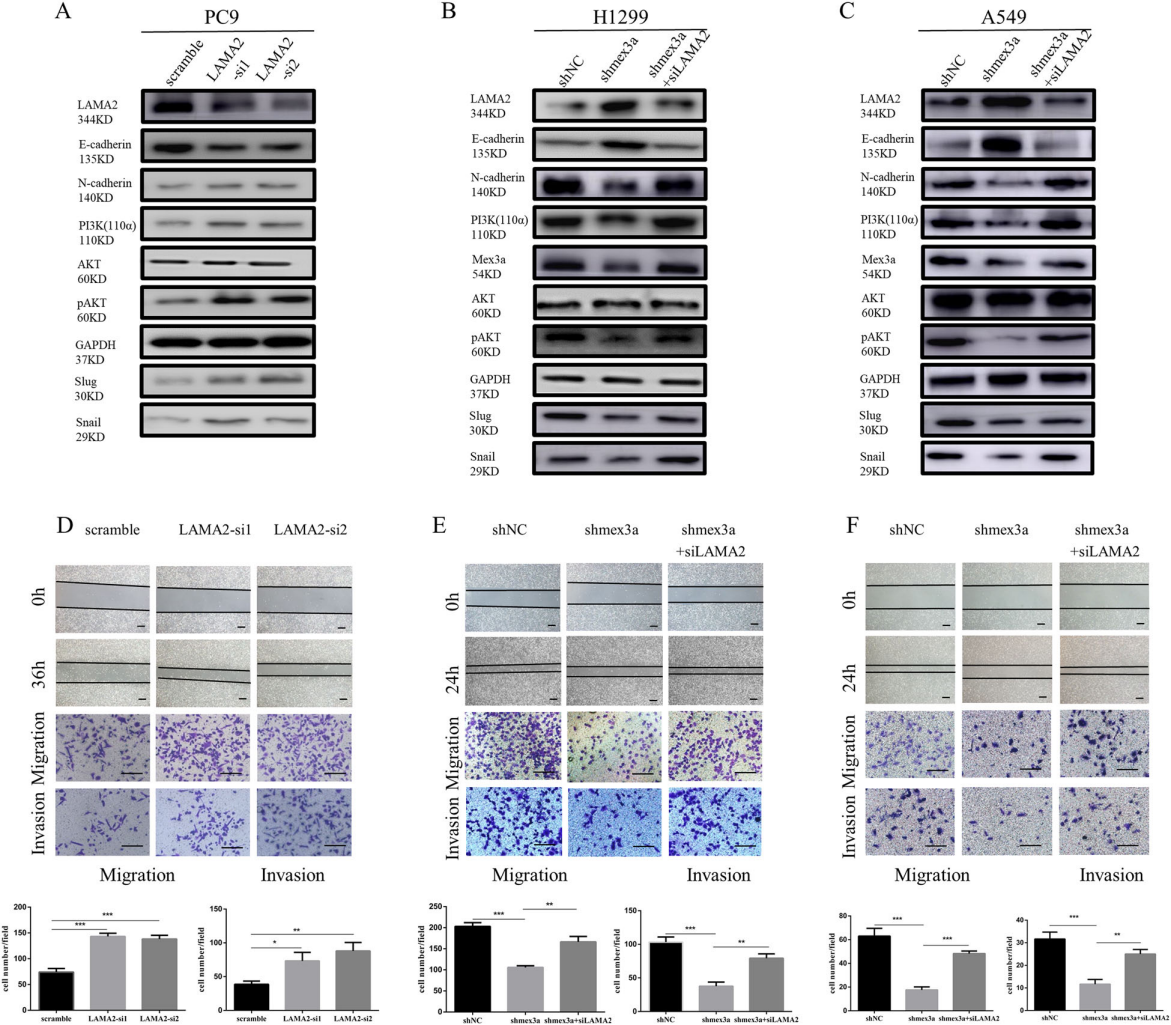
Mex3a activates the PI3K/Akt signaling pathways through LAMA2. **a** Western blot analysis showed altered levels of PI3K-AKT pathway and EMT-related markers after LAMA2 knockdown in PC9 cell by using 2 independent siRNAs. **b**, **c** Western blot analysis of PI3K/Akt signaling pathways and EMT-related markers of the rescue experiment in H1299 (**b**) and A549 cell (**c**). **d** Wound healing, migration, and invasion assays were performed to identify metastasis ability after LAMA2 knockdown in PC9 cell. Data are presented as the mean ± S.D. *n* = 3. **e**, **f** Wound healing, migration, and invasion assays of the rescue experiment in H1299 (**e**) and A549 cell (**f**). Data are presented as the mean ± S.D. *n* = 3. *P < 0.05, **P < 0.01, ***P < 0.001, and ****P < 0.0001.

Among LUAD cells, PC9 cell expressed higher LAMA2 with lower Mex3a (Supplementary Fig. 1C, K). We established LAMA2 knockdown PC9 cell with two sequences of siRNA (Supplementary Fig. 1L). Knockdown of LAMA2 promoted migration, invasion, EMT and activation of PI3K-AKT pathway in PC9 cell (Fig. 6a, d). To verify whether LAMA2 was involved in Mex3a-mediated metastasis effects on LUAD cells, we performed a rescue experiment. We knockdown LAMA2 with simultaneous knockdown of Mex3a expression in H1299 and A549 cell, and discovered that knockdown of LAMA2 partially attenuated the decreased cell migration and invasion capacity caused by Mex3a knockdown (Fig. 6b, c, e, f). These results demonstrated that LAMA2 was an important inhibitory factor in LUAD metastasis and that LAMA2 was essential for inhibiting the metastasis ability of LUAD induced by Mex3a.

### Mex3a promoted LUAD cell metastasis through LAMA2 in vivo

**Fig. 7:**
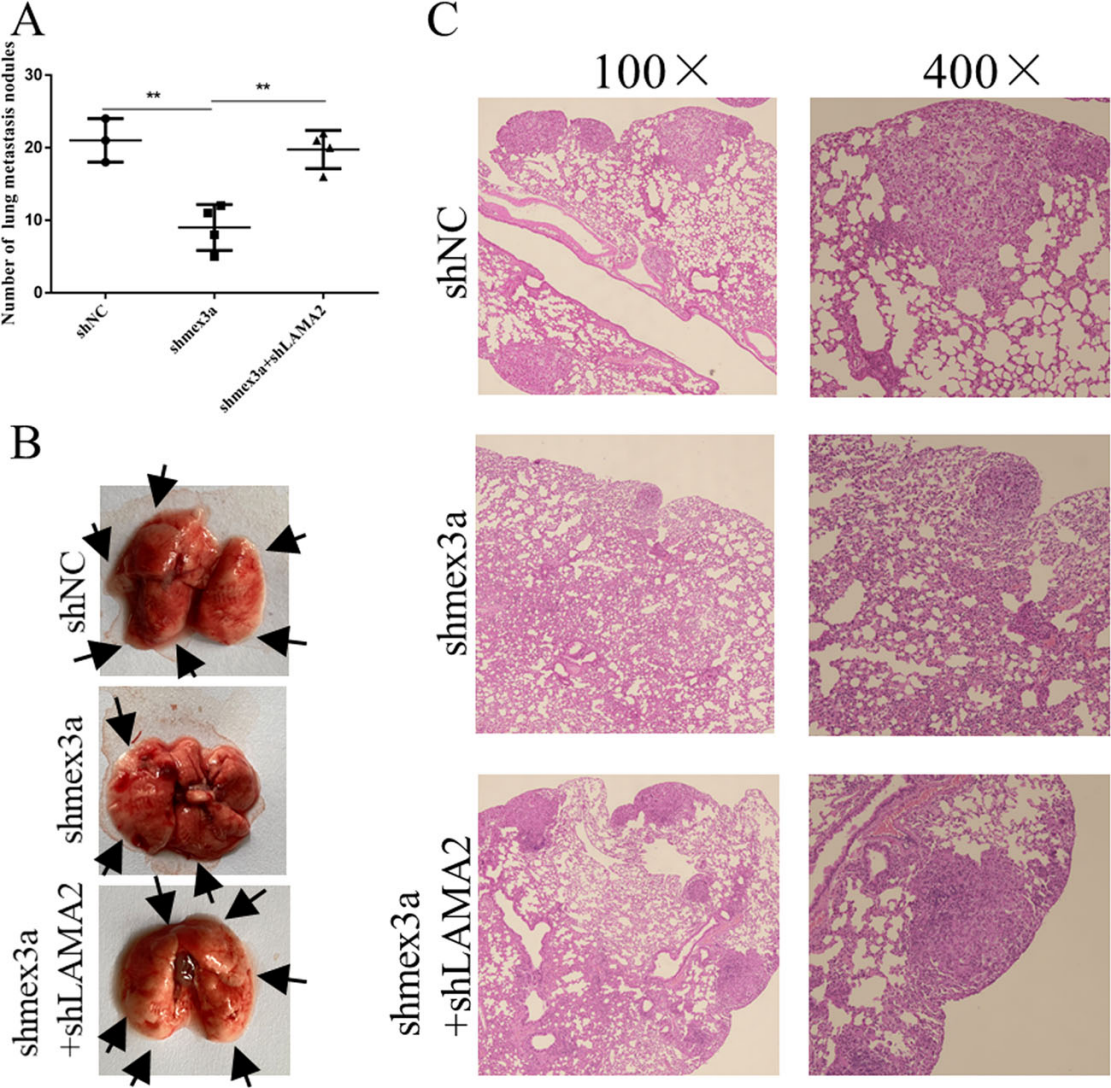
Mex3a promoted LUAD cell metastasis through LAMA2 in vivo. **a** Bar chart of lung metastasis nodules in shNC, shmex3a and shmex3a+shLAMA2 groups. **b** Representative images of lung metastasis. **c** Representative images of HE staining of lung metastasis. *P < 0.05, **P < 0.01, ***P < 0.001, and ****P < 0.0001.

After verifying the function of LAMA2 in vitro, we would like to confirm the function of LAMA2 in vivo. LAMA2 knockdown increased the number of metastasis lesions in Mex3a-knockdown group (Fig. 7a, b). Tumor metastasis was confirmed by HE staining (Fig. 7c). In conclusion, both in vitro and in vivo data proved Mex3a promoted LUAD cell metastasis through LAMA2.

## Discussion

This study highlights RBP Mex3a’s function and mechanisms in regulating LUAD metastasis. In the process of cancer, RBP regulates the expression of many important genes post-transcriptionally by altering its encoded mRNA. Previous studies have shown that Mex3a regulates CDX2 in human colon cancer and promoted proliferation in gastric cancer and glioblastoma^2,4,18^. Here, for the first time, we identified that Mex3a was upregulated in human LUAD and high Mex3a expression correlated with positive lymph node metastasis, and advanced TNM stage, suggesting that Mex3a can be a strong predictor for LUAD metastasis and prognosis. In addition, we found that Mex3a promoted the proliferation of primary tumors in vitro and vivo. Taken together, Mex3a can be a promising potential biomarker for prognosis and therapeutic target in LUAD.

Our research also demonstrated that Mex3a facilitated LUAD metastasis through regulation of many genes associated with ECM-receptor interaction. Integrative analysis of transcriptome sequence identified at least LAMA2, CLDN1, PIK3R3, and COL5A1 as Mex3a possible targets. However, it was not reliable to discover the target of Mex3a by transcriptome sequencing. Technically, combination of transcriptome sequencing, RIP sequencing and transcript stability sequencing should be performed to search targets and identify function (mRNA decay, translation, or others) of RNA-binding protein. Fortunately, RIP result confirmed Mex3a can directly bind to LAMA2 mRNA and Mex3a facilitated LAMA2 mRNA instability in actinomycin experiments. In this study, we identified LAMA2 as the most highly differentially expressed gene associated with ECM-receptor interaction as result of Mex3a knockdown, and we observed that LAMA2 was downregulated and correlated with poor clinical outcomes in LUAD. In addition, LAMA2 inhibited LUAD metastasis. We then described the strong negative correlation between Mex3a and LAMA2 expression in LUAD tissues. In addition, knockdown of LAMA2 can partially attenuate the decline in cell migration and invasion caused by mex3a knockdown, providing sufficient evidence that LAMA2 is Mex3a target gene.

Our work demonstrated LAMA2 exerted a suppressive-properties in LUAD. Work by Beltran et al. showed that suppression of laminin expression promoted the invasiveness of one breast cancer cell line^19^. What’s more, lower expression levels of LAMA2 predict poor survival and higher chance of cancer recurrence in HCC patients, suggesting an important role of the extracellular matrix and cell adhesion in tumor progression of a subgroup of HCC patients^20^. Whole-exome and whole-genome sequencing discovered olfactory neuroblastoma and breast cancer has a deletion in LAMA2^21,22^. The first exon of LAMA2 is full of CpG islands, which have been reported as hypermethylated in multiple cancers^23^. And promoter hypermethylation is often associated with decreased expression of tumor suppressor genes^24^. LAMA2 expression were decreased in drug resistance ovarian cancer cell line^25,26^. However, recent study have revealed opposite effects of LAMA2 as an oncoprotein in glioblastoma and ependymoma^27,28^. We thought the function of LAMA2 may be tumor specific or dependent on the stage of oncogenesis.

Our study also demonstrated that Mex3a could activate the PI3K-AKT signaling pathways in LUAD. Accumulating evidence revealed that intracellular PI3K-AKT pathway are key signaling mediators to activate the EMT inducing transcription factors^29^. A number of key transcription factors, including Twist, Snail, Slug, Zinc-finger E-box-binding (ZEB) family, have been described as major drivers of the EMT program^30^. Claudin-1 is a direct downstream target gene of Snail and Slug^31^. The Transcriptome sequencing result revealed change of EMT-related genes (N-cadherin, CLDN1, and Snail) after Mex3a knockdown. We observed the upregulation of E-cadherin, which was associated with and Snail, Slug and N-cadherin downregulation after Mex3a knockdown in protein level. And LAMA2 knockdown promoted the activation of PI3K-AKT pathway, which is consistent with former study^13^. Moreover, LAMA2 knockdown compromised the inactivation caused by Mex3a depletion. However, we cannot rule out other pathways regulated by Mex3a due to the limited detection methods, considering the significantly critical roles of Mex3a in LUAD. Additionally, LAMA2 LG1 domain could mediate cell attachment through syndecan-1, which is necessary to maintain the epithelial phenotype^32,33^. We considered LAMA2 also can exert anchoring function to maintain normal morphogenesis to prevent metastasis ability. This idea should be further confirmed.

However, there are still several questions to be addressed as follows: (1) How does Mex3a prevent expression mRNA of LAMA2? Four Mex3 proteins all include similar KH domain and finger domain^1^. Karine Buchet–Poyau first reported Mex3 family as a RNA-binding protein and Mex3a colocalize with both the decapping enzyme hDcp1a and Ago1 proteins in P bodies^1^. Evidence suggests that P body-associated mRNA decapping are translationally repressed and can be degraded or stored for subsequent translation^34^. Cano summarized Buchet–Poyau and his findings, and proposed a model of mRNA degradation mediated by Mex3c^35^. Considering similar domain, we proposed Mex3a protein could recognize and bind to the target mRNA through two KH domains, and then the Mex3a bound to the target mRNA interacts with the Argonaute protein, and then the target mRNA was degraded. We will focus the hypothesis in our future work. (2) What precise genomic target sites of LAMA2 can Mex3a identify? Mex3c bound in the 3’-UTR of the human leukocyte antigen serotype (HLA-A2) mRNA with specific RNA-binding motif through KH domain^36^. We found the similar binding motif located in 5’-UTR, CDS and 3’-UTR of LAMA2 mRNA. However, the sequence of LAMA2 mRNA was too long (up to 9000 bp) to construct proper plasmid to perform RNA-pulldown assay or dual-luciferase assay. We still should search appropriate method to discover the binding region of Mex3a in future.

In summary, we report that Mex3a, a novel protumorigenic RBP in LUAD, can promote cell metastasis through the PI3K/AKT signaling pathway mediated by LAMA2, ultimately affecting survival in LUAD patients. Our findings underscore the crucial roles of Mex3a in LUAD metastasis and its potential prognostic and therapeutic value.

## Materials and methods

### Human LUAD samples and cell lines

We collected seven-paired LUAD and para-tumor tissues to perform microarray sequencing, 94-paired LUAD and para-tumor tissues to construct tissue microarrays. LUAD and para-tumor tissues were surgically resected from LUAD patients at QiLu Hospital (Jinan, China) between 2007 and 2014. All diagnosis were confirmed by pathology. Complete clinicopathological and follow-up data for the 101 paired LUAD samples were available. All experiments were approved and supervised by the Medical Ethics Committee of Qilu Hospital of Shandong University (KYLL-2016-097).

The LUAD cell lines HBE, A549, H1299, PC9, HCC827, and H1975 were purchased from Institute of Cell Research, Shanghai Cell Bank, Chinese Academy of Sciences, and cultured by regular 1640 medium. All cell lines were had STR profiling and were tested every 3 months for mycoplasma contamination. Cells were transiently transfected with siRNAs or plasmids using jetPRIME transfection reagent (Polyplus, USA), following the manufacturer’s protocol. After 48–72 h transfection, cells were collected and lysed to evaluate the transfection efficiency. Mex3a overexpression and knockdown lentiviruses were purchased from Guangzhou Genecopia Company. PC9 stable cell lines overexpressing Mex3a were infected with lentivirus (MOI: 20) and selected with 0.5 mg/ml puromycin for about 1 week. H1299 and A549 stable cell lines lacking Mex3a were infected with lentivirus (MOI: 10 and 20, respectively) and selected with 1 mg/ml puromycin for about 1 week. H1299 stable cell line lacking LAMA2 was selected with 2 ug/ml blasticidin for about 1 week. siRNAs sequences are listed in supplementary data.

### CCK8 and EDU assay

For CCK8 and EDU analysis, cells were transfected with Mex3a siRNA or plasmid. Cell viability was detected with the Cell Counting Kit-8 (CCK8) (APExBIO, #K1018) following the manufacturer’s protocol. In a 96-well plate, 100 μL of cell suspension at a density of 2 × 10^3^ cells per well was seeded in each well, and precultured in a cell incubator for 24 h. Proliferation rates were detected at 0, 24, 48, and 72 h after transfection, and absorbance reading was performed on a microtiter plate reader at 450 nm following the manufacturer’s protocol.

For EDU (RiboBio, #C10310) analysis, first cells were cultured in 10 mol/L EDU -containing medium for 4 h. Then cells were fixed by 4% paraformaldehyde at room temperature for 60 min after removing EDU-containing medium. Images were acquired using a fluorescence microscope after washing and Apollo and Hoechst dye following the manufacturer’s protocol. The percentage of EDU positive cells is calculated as follows: (EDU stained cells/Hoechst stained cells) × 100%.

### Wound healing, migration, and invasion assay

To perform wound healing, we seeded moderate cells in six-well plates. The inoculation principle is 100% fusion rate after overnight. Scrap the cell layer at the bottom of the well with a sterile 1000 μl pipette tip to create a linear gap. At 24 and 36 h, plates were taken with an inverted fluorescence microscope to acquire images. The rate of closure of the open wounds was calculated. Scratch healing rate = (healing width at 24 h or 36 h-healing width at 0 h)/healing width at 0 h.

Migration assays were performed using trans-well chambers in 24-well plates (BD Biosciences, #353092). In brief, LUAD cells with different treatments were seeded in the upper chambers suspended with 100 μl serum-free medium, the lower chambers were full of 700 μl 1640 containing 20% FBS. After incubated at 37 °C for 48 h, the cells on the upper surface of the membrane were removed by using cotton swab. The migrated cells on the lower side of the filter were fixed with 4% paraformaldehyde at room temperature for 60 min and stained with 0.1% crystal violet dye for 60 min, and the number of cells migrating to the lower surface was counted in three randomly selected high-magnification fields for each sample. For invasion assays, we paved Matrigel (BD, biocoat, #358248) in the surface of upper chamber. The remaining step was same as migration assays.

### Immunohistochemistry

LUAD tissue and mouse tumor tissue were fixed by 4% paraformaldehyde at room temperature for 60 min. After incubated at 62 °C for 2 h, dewaxed and rehydrated, antigen extraction was performed using citrate buffer (pH 6) at 97 °C for 20 min. 3% hydrogen peroxide was used to block endogenous peroxidase activity for 10 min at room temperature. Nonspecific binding of antibodies can be prevented by incubating slides with 5% normal goat serum in PBST for 1 h at room temperature. Slides were then incubated with primary antibodies against, Ki67, E-cadherin, N-cadherin, and LAMA2 at 4 °C overnight. After washing three times with TBST, each slide was incubated with the appropriate HRP-labeled secondary antibody and the signal was developed with DAB solution before counterstaining with hematoxylin. Image Pro-Plus (IPP) was used to analyze the intensity of immunohistochemical staining.

### RNA extraction and RT-PCR

Total RNA was extracted from cells with different treatment by using Trizol reagent according to the manufacturer’s instructions. cDNA was then synthesized using the SureScript First-Strand cDNA Synthesis Kit (Genecopia, #QP056). RT-PCR analysis was carried on BioAnalyzer 2100 system (Agilent Technologies, Inc, USA). Primer sequences are listed in supplementary data (List of primer sequences).

### Western blotting

Cells and LUAD samples were harvested in RIPA buffer (Beyotime, Shanghai, China) and centrifuged for 10 min at 12,000 × *g* and 4 °C. Then supernatants were collected, and protein concentrations were calculated by the BCA Kit (Ysasen, Shanghai, #B68010). We took 40–60 μg per sample, and performed protein separation by SDS-PAGE electrophoresis and transferred gel onto PVDF membrane. After blocked by the 5% milk powder at room temperature for 2 h, the membranes were incubated with primary antibodies against Mex3a (Abcam, #ab79046), GAPDH (Cell Signaling Technology, #5174), LAMA2 (Abcam, #ab236762), E-cadherin (Cell Signaling Technology, #14472), N-cadherin(Cell Signaling Technology, #13116), Snail (Cell Signaling Technology, #3879), Slug(Cell Signaling Technology, #9585), PI3K p110α (Cell Signaling Technology, #4249), pAKT (Cell Signaling Technology, #4060), and AKT (Cell Signaling Technology, #4691) at 4 °C overnight. The membrane was incubated with a secondary antibody at room temperature for 1 h After three washes with TBST. Then, the signals were detected by enhanced chemiluminescence following the manufacturer’s recommendations.

### *m*RNA microarray, Transcriptome sequencing and bioinformatics analysis

Microarray experiments were performed by KangCheng Bio-tech, Shanghai, China. The Agilent Whole Genome Oligo Microarray was used to identify mRNA transcripts with differential expression between LUAD and adjacent normal tissues. Tissue samples were used for the array analysis according to the manufacturer’s protocol. Related data was uploaded in GEO datasets (GSE 140797).

Genome-wide transcriptional sequencing were performed by Baimaike, Beijing, China. Transcriptome sequencing (NEB, USA) was used to identify mRNA transcripts with differential expression between Mex3a-silenced H1299 cells and control H1299 cells. The RNA preparation and Library preparation for Transcriptome sequencing were performed according to the manufacturer’s instructions.

The gene expression and clinical data of LUAD patients from the Cancer Genome Atlas (TCGA) were downloaded from UCSC Xena Browser (https://xenabrowser.net/). The gene expression and clinical data of LUAD patients from the GEO datasets were obtained using GSE 116959 and GSE 19804 (https://www.ncbi.nlm.nih.gov/gds).

### RNA immunoprecipitation (RIP) assay

RNA immunoprecipitation assays were carried out using a Magna RIP Kit (Millipore, #17-701) following the manufacturer’s instructions. Briefly, for Mex3a RIP, PC9 cells were transfected with Mex3a overexpression plasmids, which was inserted in flag tag. After 48 h, the cells were harvested to perform RIP experiments using an anti-flag antibody (Cell Signaling Technology, #14793) or IgG antibody (Millipore, #17-701). Following the recovery of antibodies using protein A/G beads, RT-PCR was performed on the precipitates to detect expression of Mex3a mRNA.

### mRNA stability assay

Cells with different treatment were incubated for the indicated times following the addition of 5 μg/mL actinomycin D (MCE, #HY-17559).

### Animal experiment

All animal experiments were approved by the Medical Ethics Committee of Shandong University. BALB/c mice (male, 6–8 weeks of age) were used in animal experiment. Animals were separated randomly. The investigators were blinded to the group allocation during the experiment and when assessing the outcome. For subcutaneous xenograft experiments (*n* = 5), H1299 cell line (3 × 10^6^ cells/mice) with stable knockdown Mex3a and control H1299 cells were subcutaneously injected into the nude mice. The size of the tumors was measured by Vernier calliper once every 5 days, and tumor volumes were calculated using the following formula: 1/2 × *d*^2^ × *D*. The mice were sacrificed after 4 weeks, and tumors were removed for assessment.

For the in vivo metastasis assay (*n* = 5), H1299 cell line (3 × 10^5^ cells/mice) with stable Mex3a knockdown and control H1299 cells were injected into the caudal vein of each nude mouse. Mouse weights were measured every week. The mice were sacrificed after 7 weeks, and the number of nodules on the lungs was confirmed by HE staining and counted.

### Statistical analysis

All statistical analyses were performed using GraphPad Prism Software and R software (version 3.6.1). Each assay was performed in at least three independent replicates. For comparisons, Student’s *t*-test (two-sided), nonparametric Mann–Whitney test, Wilcoxon signed-rank test, Pearson’s Chi-square test, log-rank test, Kaplan–Meier survival analysis, Fisher’s exact test, and Pearson’s correlation analysis were performed as indicated. One-way ANOVA was used to compare the differences among more than two groups.

## Supplementary information

Supplementary Figure 1

Supplementary material 1

Supplementary material 2
