# Supplementary figures and images for: Mex3a interacts with LAMA2 to promote lung adenocarcinoma metastasis via PI3K/AKT pathway
Source: Cell Death Dis. 2020 Aug 13;11(8):614. doi: 10.1038/s41419-020-02858-3 (PMC7427100; doi:10.1038/s41419-020-02858-3)

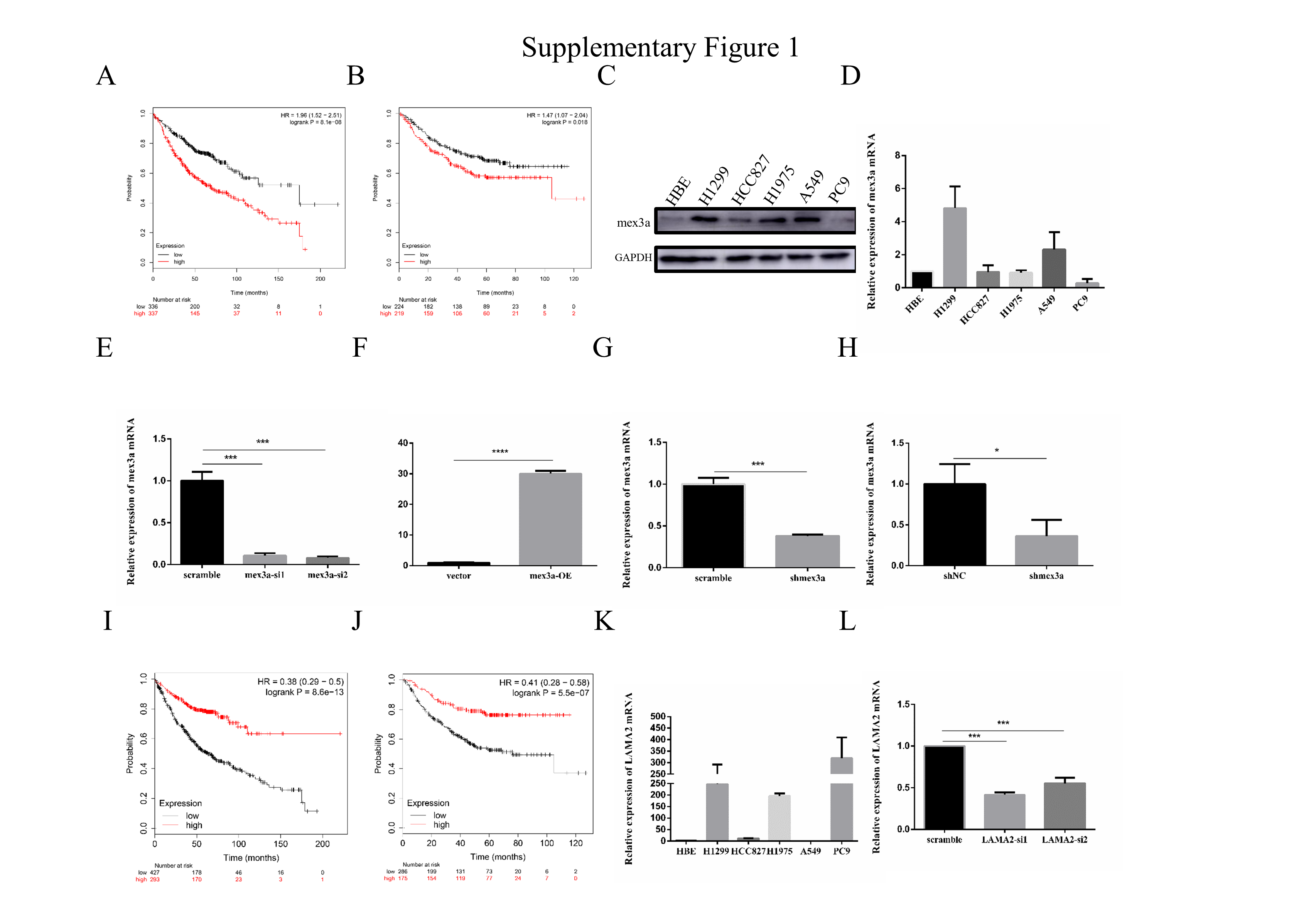

Supplement: Supplementary file 1 — Supplementary Figure 1 [file 41419_2020_2858_MOESM1_ESM.tif]
