# Supplementary material for: Mex3a interacts with LAMA2 to promote lung adenocarcinoma metastasis via PI3K/AKT pathway
Source: Cell Death Dis. 2020 Aug 13;11(8):614. doi: 10.1038/s41419-020-02858-3 (PMC7427100; doi:10.1038/s41419-020-02858-3)
Supplement: Supplementary file 2 — Supplementary material 1 [file 41419_2020_2858_MOESM2_ESM.docx]

Table 1

| SiRNA sequence | sense（5'-3'） | antisense（5'-3'） |
| --- | --- | --- |
| Scramble | UUCUCCGAACGUGUCACGUTT | ACGUGACACGUUCGGAGAATT |
| mex3a-si1 | GCAAGAUCCUCGAGUACAATT | UUGUACUCGAGGAUCUUGCTT |
| mex3a-si2 | GCGGAGUGGACUCUGGCUUTT | AAGCCAGAGUCCACUCCGCTT |
| LAMA2-si1 | GGUAUAAACUGCGAGACAUTT | AUGUCUCGCAGUUUAUACCTT |
| LAMA2-si2 | GGUAAUAAUUGUGACCCAATT | UUGGGUCACAAUUAUUACCTT |
