# Supplementary material for: Mex3a interacts with LAMA2 to promote lung adenocarcinoma metastasis via PI3K/AKT pathway
Source: Cell Death Dis. 2020 Aug 13;11(8):614. doi: 10.1038/s41419-020-02858-3 (PMC7427100; doi:10.1038/s41419-020-02858-3)
Supplement: Supplementary file 3 — Supplementary material 2 [file 41419_2020_2858_MOESM3_ESM.docx]

Table 2

| Primer sequence | Forward | Reverse |
| --- | --- | --- |
| Mex3a | TGGAGAACTAGGATGTTTCGGG | GAGGCAGAGTTGATCGAGAGC |
| GAPDH | GGAGCGAGATCCCTCCAAAAT | GGCTGTTGTCATACTTCTCATGG |
| LAMA2 | TGCTGTCCTGAATCTTGCTTC | AGCATTTGTAATCGGGTGTCTC |
| CLDN1 | CCTCCTGGGAGTGATAGCAAT | GGCAACTAAAATAGCCAGACCT |
| ITGA2B | GATGAGACCCGAAATGTAGGC | GTCTTTTCTAGGACGTTCCAGTG |
| ITGA7 | CAGCGAGTGGACCAGATCC | CCAAAGAGGAGGTAGTGGCTATC |
| DDIT4 | TGAGGATGAACACTTGTGTGC | CCAACTGGCTAGGCATCAGC |
| SPP1 | CTCCATTGACTCGAACGACTC | CAGGTCTGCGAAACTTCTTAGAT |
| SHC2 | TCCTACGTCGTGCGGTACAT | CCTCATGGAGCCGGTTGATG |
| PCK2 | AGTAGAGAGCAAGACGGTGAT | TGCTGAATGGAAGCACATACAT |
| DCHS1 | GACATCAACGACCATGCTCCA | GTGCCAAAAGCTGTATGCTCA |
| PIK3R3 | TACAATACGGTGTGGAGTATGGA | TACAATACGGTGTGGAGTATGGA |
| COL5A1 | GCCCGGATGTCGCTTACAG | AAATGCAGACGCAGGGTACAG |
| COL6A3 | ATGAGGAAACATCGGCACTTG | GGGCATGAGTTGTAGGAAAGC |
| PDGFA | GCAAGACCAGGACGGTCATTT | GGCACTTGACACTGCTCGT |
| ITGA10 | AACATCACCCACGCCTATTCC | GTTGGTAGTCACCTAAGTGGC |
| LAMA4 | GTAATGCCTACTTTACCAGGGT | GGGAGTTTCAGAGCAACAGG |
| ITGB3 | CATGAAGGATGATCTGTGGAGC | AATCCGCAGGTTACTGGTGAG |
| LAMA3 | CACCGGGATATTTCGGGAATC | AGCTGTCGCAATCATCACATT |
| ITGA2 | CCTACAATGTTGGTCTCCCAGA | AGTAACCAGTTGCCTTTTGGATT |
